# Supplementary material for: PTC2 region genotypes counteract Biomphalaria glabrata population differences between M-line and BS90 in resistance to infection by Schistosoma mansoni
Source: PeerJ. 2022 Sep 13;10:e13971. doi: 10.7717/peerj.13971 (PMC9480060; doi:10.7717/peerj.13971)
Supplement: Supplemental Information 1 [file peerj-10-13971-s001.docx]

**Relevant code used for analyses and figures:**

Logistic regressions were done using the SYSTAT 13.2 package with default settings. Example commands are with the detailed results in supplemental table S3.

The Dot plot in Fig 6 was generated with ChopFastaStaggered.pl and AssessBlatChopped.pl (available at [https://github.com/jacobtennessen/MiSCVARS/](https://nam04.safelinks.protection.outlook.com/?url=https%3A%2F%2Fgithub.com%2Fjacobtennessen%2FMiSCVARS%2F&data=05%7C01%7CMichael.Blouin%40oregonstate.edu%7Cdf1ea60980aa455b184e08da69a23228%7Cce6d05e13c5e4d6287a84c4a2713c113%7C0%7C0%7C637938443468935650%7CUnknown%7CTWFpbGZsb3d8eyJWIjoiMC4wLjAwMDAiLCJQIjoiV2luMzIiLCJBTiI6Ik1haWwiLCJXVCI6Mn0%3D%7C3000%7C%7C%7C&sdata=1xKT1GAFcugYzGwO7PHlXxNcrlvqHaxJoF7KwUbl85o%3D&reserved=0)) along with BLAT (Kent, 2002; stepSize = 1), as in Tennessen et al. (2020). The reference is Kent WJ (2002) BLAT--the BLAST-like alignment tool. Genome Research 12:656–664.[https://doi.org/10.1101/gr.229202](https://nam04.safelinks.protection.outlook.com/?url=https%3A%2F%2Fdoi.org%2F10.1101%2Fgr.229202&data=05%7C01%7CMichael.Blouin%40oregonstate.edu%7Cdf1ea60980aa455b184e08da69a23228%7Cce6d05e13c5e4d6287a84c4a2713c113%7C0%7C0%7C637938443468935650%7CUnknown%7CTWFpbGZsb3d8eyJWIjoiMC4wLjAwMDAiLCJQIjoiV2luMzIiLCJBTiI6Ik1haWwiLCJXVCI6Mn0%3D%7C3000%7C%7C%7C&sdata=g6MrmhlDZKSFoya9vHUOa9ZoBzMxcKwYdJz%2FEr3eQKI%3D&reserved=0)

The FLYE assembly was run using the following settings:

flye --pacbio-raw {sample_pacbio-raw.datafile} -g 1g -i 2 --asm-coverage 50 -t 16 -o flye/{sample}
