# Supplementary material for: PTC2 region genotypes counteract Biomphalaria glabrata population differences between M-line and BS90 in resistance to infection by Schistosoma mansoni
Source: PeerJ. 2022 Sep 13;10:e13971. doi: 10.7717/peerj.13971 (PMC9480060; doi:10.7717/peerj.13971)
Supplement: Supplemental Information 2 [file peerj-10-13971-s002.docx]

Table S1. Primer sequences (5’ to 3’), locations and amplicon sizes.

| Locus | Primer Forward | Primer Reverse | amplicon size BS90 | amplicon size M-line |
| --- | --- | --- | --- | --- |
| *up2* | AGTAACAACAGTAAACGTAACTTTCT | CCAGTACTGCGATTGGTTAGGGG | 928 | 503 |
| *up1* | TCATTTATCCCTTAGTCTGGTGG | CAAGACCCATGCGATCACAG | 880 | 485 |
| *0* | ATAGAATGATATAGTGGAGCTTGCA | GATCCAGGAATTTGTTTCGGAAGTA | 751 | 979 |
| *dn1* | GGCAGACAATCTTGAGACAGTAG | CTAGTCAAGAACAAGAGCGAAAG | 596 | 213 |
| *dn2* | CATGCAATAACTAACCTGATGCCAA | GTAACTGATCACGAGAGGGAAAACC | 1284 | 1086 |
| *OPM-04* | GTCCTAGTTTCACGGGTCTTTTCCAC | CTAAATCTGGGCAGTTGAACGTGAG | 413 | 417/422 |

Locations of each primer in the BS90 and M-line assembly. Each cell shows the contig/scaffold on which each locus is found in each assembly, and then the locations of first the Forward and then the Reverse primers.

| Locus | positions in BS90 assembly | positions in M-line assembly |
| --- | --- | --- |
| *up2* | scaffold_1992: 1796249-1796274; 1797176-1797154 | scaffold_445: 9514776-9514751; 9514274-9514296 |
| *up1* | contig_846: 3941323-3941301; 3940444-3940463 | scaffold_445: 8325239-8325217; 8324755-8324774 |
| *0* | contig_846: 3017461-3017437; 3016711-3016735 | scaffold_445: 7134248-7134224; 7133270-7133294 |
| *dn1* | contig_846: 1471779-1471757; 1471184-1471206 | scaffold_445: 5292940-5292918; 5292728-5292750 |
| *dn2* | contig_844: 2739581-2739557; 2738298-2738322 | scaffold_445: 3224665-3224641; 3223580-3223604 |
| *OPM-04* | contig_2117: 382604-382579; 382192-382216 | scaffold_445: 11758333-11758358; 11758749-11758725 |
