# Supplementary material for: PTC2 region genotypes counteract Biomphalaria glabrata population differences between M-line and BS90 in resistance to infection by Schistosoma mansoni
Source: PeerJ. 2022 Sep 13;10:e13971. doi: 10.7717/peerj.13971 (PMC9480060; doi:10.7717/peerj.13971)
Supplement: Supplemental Information 3 — The raw counts of infected and not-infected individuals used for the graphs and logistic regression analyses from each experiment. [file peerj-10-13971-s003.docx]

Table S2. Raw genotype and infection data used for logistic regressions and figures.

“B into M” populations, locus *PTC2*

| population | *PTC2* genotype | infected?(1=yes) | number observed |
| --- | --- | --- | --- |
| M8.2 | BB | 1 | 8 |
|  | BB | 0 | 14 |
|  | BM | 1 | 13 |
|  | BM | 0 | 79 |
|  | MM | 1 | 5 |
|  | MM | 0 | 40 |
| M8-2.2 | BB | 1 | 15 |
|  | BB | 0 | 4 |
|  | BM | 1 | 31 |
|  | BM | 0 | 19 |
|  | MM | 1 | 9 |
|  | MM | 0 | 11 |
| M13.2 | BB | 1 | 10 |
|  | BB | 0 | 11 |
|  | BM | 1 | 17 |
|  | BM | 0 | 28 |
|  | MM | 1 | 6 |
|  | MM | 0 | 13 |
| M17 | BB | 1 | 8 |
|  | BB | 0 | 4 |
|  | BM | 1 | 30 |
|  | BM | 0 | 11 |
|  | MM | 1 | 11 |
|  | MM | 0 | 4 |

“M into B” population, locus *PTC2*

| population | | *PTC2* genotype | infected?(1=yes) | | | number observed | |
| --- | --- | --- | --- | --- | --- | --- | --- |
|  |  | | |  |  | |  |
| B7.2 | BB | | | 1 | 3 | |  |
| B7.2 | BB | | | 0 | 30 | |  |
| B7.2 | BM | | | 1 | 7 | |  |
| B7.2 | BM | | | 0 | 57 | |  |
| B7.2 | MM | | | 1 | 1 | |  |
| B7.2 | MM | | | 0 | 18 | |  |

F2 entire sample, N = 329, locus *PTC2*

| population | *PTC2* genotype | infected?(1=yes) | number observed |
| --- | --- | --- | --- |
| F2(N=329) | BB | 0 | 69 |
|  | BB | 1 | 24 |
|  | BM | 0 | 140 |
|  | BM | 1 | 29 |
|  | MM | 0 | 58 |
|  | MM | 1 | 9 |

F2 cases & controls, locus *PTC2*

| population | *PTC2* genotype | infected?(1=yes) | number observed |
| --- | --- | --- | --- |
| F2(case/control) | BB | 0 | 10 |
|  | BB | 1 | 23 |
|  | BM | 0 | 36 |
|  | BM | 1 | 24 |
|  | MM | 0 | 9 |
|  | MM | 1 | 7 |

F2 cases & controls, locus *OPM-04*

| population | *OPM-04* genotype | infected?(1=yes) | number observed |
| --- | --- | --- | --- |
| F2(case/control) | BB | 0 | 8 |
|  | BB | 1 | 13 |
|  | BM | 0 | 28 |
|  | BM | 1 | 27 |
|  | MM | 0 | 16 |
|  | MM | 1 | 15 |
