# Supplementary material for: PTC2 region genotypes counteract Biomphalaria glabrata population differences between M-line and BS90 in resistance to infection by Schistosoma mansoni
Source: PeerJ. 2022 Sep 13;10:e13971. doi: 10.7717/peerj.13971 (PMC9480060; doi:10.7717/peerj.13971)
Supplement: Supplemental Information 4 [file peerj-10-13971-s004.docx]

Table S3. Binary logistic regression output.

Results were obtained using the data in supplemental table S2, via the BLOGIT command in SYSTAT 13.2. Below are sample commands to analyze a single population, where in this example DOSEM = number of M alleles (0, 1 or 2 for the additive model):

REM -- Following commands were produced by the BLOGIT dialog:

BLOGIT

MODEL INFECTED = CONSTANT+DOSEM / REFER = 1.000

ESTIMATE / CONFI = 0.95 ITER = 50

REM -- End of commands from the BLOGIT dialog

**“B into M” populations, locus *0***

Additive model. AIC = 458.78

| **Parameter Estimates** | | | | | | |
| --- | --- | --- | --- | --- | --- | --- |
| **Parameter** | **Estimate** | **Standard Error** | **Z** | **p-Value** | **95% Confidence Interval** | |
|  |  |  |  |  | **Lower** | **Upper** |
| CONSTANT | 0.810 | 0.397 | 2.041 | 0.041 | 0.032 | 1.587 |
| GENOTYPE | 0.790 | 0.352 | 2.246 | 0.025 | 0.101 | 1.480 |
| POP$_13.2 | -0.684 | 0.551 | -1.241 | 0.214 | -1.764 | 0.396 |
| POP$_17 | -1.597 | 0.650 | -2.457 | 0.014 | -2.871 | -0.323 |
| POP$_8-2.2 | -2.080 | 0.595 | -3.496 | 0.000 | -3.246 | -0.914 |
| POP$_13.2*GENOTYPE | -0.447 | 0.482 | -0.928 | 0.354 | -1.392 | 0.498 |
| POP$_17*GENOTYPE | -0.946 | 0.556 | -1.700 | 0.089 | -2.036 | 0.145 |
| POP$_8-2.2*GENOTYPE | -0.038 | 0.498 | -0.077 | 0.939 | -1.015 | 0.938 |

| **Odds Ratio Estimates** | | | | |
| --- | --- | --- | --- | --- |
| **Parameter** | **Odds Ratio** | **Standard Error** | **95% Confidence Interval** | |
|  |  |  | **Lower** | **Upper** |
| GENOTYPE | 2.204 | 0.776 | 1.106 | 4.394 |
| POP$_13.2 | 0.505 | 0.278 | 0.171 | 1.486 |
| POP$_17 | 0.202 | 0.132 | 0.057 | 0.724 |
| POP$_8-2.2 | 0.125 | 0.074 | 0.039 | 0.401 |
| POP$_13.2*GENOTYPE | 0.639 | 0.308 | 0.249 | 1.645 |
| POP$_17*GENOTYPE | 0.388 | 0.216 | 0.131 | 1.156 |
| POP$_8-2.2*GENOTYPE | 0.962 | 0.479 | 0.362 | 2.555 |

M-allele dominant model. AIC = 459.61

| **Parameter Estimates** | | | | | | |
| --- | --- | --- | --- | --- | --- | --- |
| **Parameter** | **Estimate** | **Standard Error** | **Z** | **p-Value** | **95% Confidence Interval** | |
|  |  |  |  |  | **Lower** | **Upper** |
| CONSTANT | 0.560 | 0.443 | 1.263 | 0.207 | -0.309 | 1.428 |
| POP$_13.2 | -0.464 | 0.622 | -0.746 | 0.456 | -1.684 | 0.756 |
| POP$_17 | -1.253 | 0.756 | -1.657 | 0.097 | -2.734 | 0.229 |
| POP$_8-2.2 | -1.881 | 0.716 | -2.626 | 0.009 | -3.285 | -0.477 |
| GENOTYPE | 1.329 | 0.510 | 2.605 | 0.009 | 0.329 | 2.329 |
| POP$_13.2*GENOTYPE | -0.846 | 0.721 | -1.175 | 0.240 | -2.259 | 0.566 |
| POP$_17*GENOTYPE | -1.642 | 0.852 | -1.926 | 0.054 | -3.312 | 0.029 |
| POP$_8-2.2*GENOTYPE | -0.295 | 0.797 | -0.370 | 0.711 | -1.857 | 1.267 |

| **Odds Ratio Estimates** | | | | |
| --- | --- | --- | --- | --- |
| **Parameter** | **Odds Ratio** | **Standard Error** | **95% Confidence Interval** | |
|  |  |  | **Lower** | **Upper** |
| POP$_13.2 | 0.629 | 0.391 | 0.186 | 2.129 |
| POP$_17 | 0.286 | 0.216 | 0.065 | 1.257 |
| POP$_8-2.2 | 0.152 | 0.109 | 0.037 | 0.620 |
| GENOTYPE | 3.778 | 1.928 | 1.390 | 10.270 |
| POP$_13.2*GENOTYPE | 0.429 | 0.309 | 0.104 | 1.761 |
| POP$_17*GENOTYPE | 0.194 | 0.165 | 0.036 | 1.029 |
| POP$_8-2.2*GENOTYPE | 0.744 | 0.593 | 0.156 | 3.551 |

**F2 population, entire sample (N = 329), locus *up1***

Additive model. AIC = 318.17

| **Parameter Estimates** | | | | | | |
| --- | --- | --- | --- | --- | --- | --- |
| **Parameter** | **Estimate** | **Standard Error** | **Z** | **p-Value** | **95% Confidence Interval** | |
|  |  |  |  |  | **Lower** | **Upper** |
| CONSTANT | 1.093 | 0.219 | 4.990 | 0.000 | 0.664 | 1.522 |
| GENOTYPE | 0.428 | 0.210 | 2.041 | 0.041 | 0.017 | 0.840 |

| **Odds Ratio Estimates** | | | | |
| --- | --- | --- | --- | --- |
| **Parameter** | **Odds Ratio** | **Standard Error** | **95% Confidence Interval** | |
|  |  |  | **Lower** | **Upper** |
| GENOTYPE | 1.535 | 0.322 | 1.017 | 2.315 |

M-allele dominant model. AIC = 318.53

| **Parameter Estimates** | | | | | | |
| --- | --- | --- | --- | --- | --- | --- |
| **Parameter** | **Estimate** | **Standard Error** | **Z** | **p-Value** | **95% Confidence Interval** | |
|  |  |  |  |  | **Lower** | **Upper** |
| CONSTANT | 1.056 | 0.237 | 4.456 | 0.000 | 0.592 | 1.521 |
| GENOTYPE | 0.595 | 0.296 | 2.010 | 0.044 | 0.015 | 1.174 |

| **Odds Ratio Estimates** | | | | |
| --- | --- | --- | --- | --- |
| **Parameter** | **Odds Ratio** | **Standard Error** | **95% Confidence Interval** | |
|  |  |  | **Lower** | **Upper** |
| GENOTYPE | 1.812 | 0.536 | 1.015 | 3.236 |

**F2 population, 55 cases and 55 controls subset (N=110), locus *up1***

Additive model. AIC = 150.05

| **Parameter Estimates** | | | | | | |
| --- | --- | --- | --- | --- | --- | --- |
| **Parameter** | **Estimate** | **Standard Error** | **Z** | **p-Value** | **95% Confidence Interval** | |
|  |  |  |  |  | **Lower** | **Upper** |
| CONSTANT | -0.552 | 0.326 | -1.697 | 0.090 | -1.190 | 0.086 |
| GENOTYPE | 0.678 | 0.310 | 2.186 | 0.029 | 0.070 | 1.287 |

| **Odds Ratio Estimates** | | | | |
| --- | --- | --- | --- | --- |
| **Parameter** | **Odds Ratio** | **Standard Error** | **95% Confidence Interval** | |
|  |  |  | **Lower** | **Upper** |
| GENOTYPE | 1.971 | 0.612 | 1.073 | 3.621 |

M-allele dominant model. AIC = 147.25

| **Parameter Estimates** | | | | | | |
| --- | --- | --- | --- | --- | --- | --- |
| **Parameter** | **Estimate** | **Standard Error** | **Z** | **p-Value** | **95% Confidence Interval** | |
|  |  |  |  |  | **Lower** | **Upper** |
| CONSTANT | -0.833 | 0.379 | -2.199 | 0.028 | -1.575 | -0.091 |
| MDOM | 1.206 | 0.445 | 2.710 | 0.007 | 0.334 | 2.078 |

| **Odds Ratio Estimates** | | | | |
| --- | --- | --- | --- | --- |
| **Parameter** | **Odds Ratio** | **Standard Error** | **95% Confidence Interval** | |
|  |  |  | **Lower** | **Upper** |
| MDOM | 3.339 | 1.485 | 1.396 | 7.985 |

**F2 population, 55 cases and 55 controls subset (N=110), locus *OPM-04***

Additive Model. AIC = 151.09

| **Parameter Estimates** | | | | | | |
| --- | --- | --- | --- | --- | --- | --- |
| **Parameter** | **Estimate** | **Standard Error** | **Z** | **p-Value** | **95% Confidence Interval** | |
|  |  |  |  |  | **Lower** | **Upper** |
| CONSTANT | -0.486 | 0.449 | -1.080 | 0.280 | -1.366 | 0.395 |
| GENOTYPE | 0.532 | 0.498 | 1.067 | 0.286 | -0.445 | 1.509 |

| **Odds Ratio Estimates** | | | | |
| --- | --- | --- | --- | --- |
| **Parameter** | **Odds Ratio** | **Standard Error** | **95% Confidence Interval** | |
|  |  |  | **Lower** | **Upper** |
| GENOTYPE | 1.702 | 0.849 | 0.641 | 4.522 |

M-allele dominant model. AIC = 151.47

| **Parameter Estimates** | | | | | | |
| --- | --- | --- | --- | --- | --- | --- |
| **Parameter** | **Estimate** | **Standard Error** | **Z** | **p-Value** | **95% Confidence Interval** | |
|  |  |  |  |  | **Lower** | **Upper** |
| CONSTANT | -0.327 | 0.366 | -0.895 | 0.371 | -1.044 | 0.389 |
| GENOTYPE | 0.247 | 0.282 | 0.877 | 0.380 | -0.306 | 0.801 |

| **Odds Ratio Estimates** | | | | |
| --- | --- | --- | --- | --- |
| **Parameter** | **Odds Ratio** | **Standard Error** | **95% Confidence Interval** | |
|  |  |  | **Lower** | **Upper** |
| GENOTYPE | 1.281 | 0.361 | 0.737 | 2.227 |
