# Supplementary figures and images for: PTC2 region genotypes counteract Biomphalaria glabrata population differences between M-line and BS90 in resistance to infection by Schistosoma mansoni
Source: PeerJ. 2022 Sep 13;10:e13971. doi: 10.7717/peerj.13971 (PMC9480060; doi:10.7717/peerj.13971)

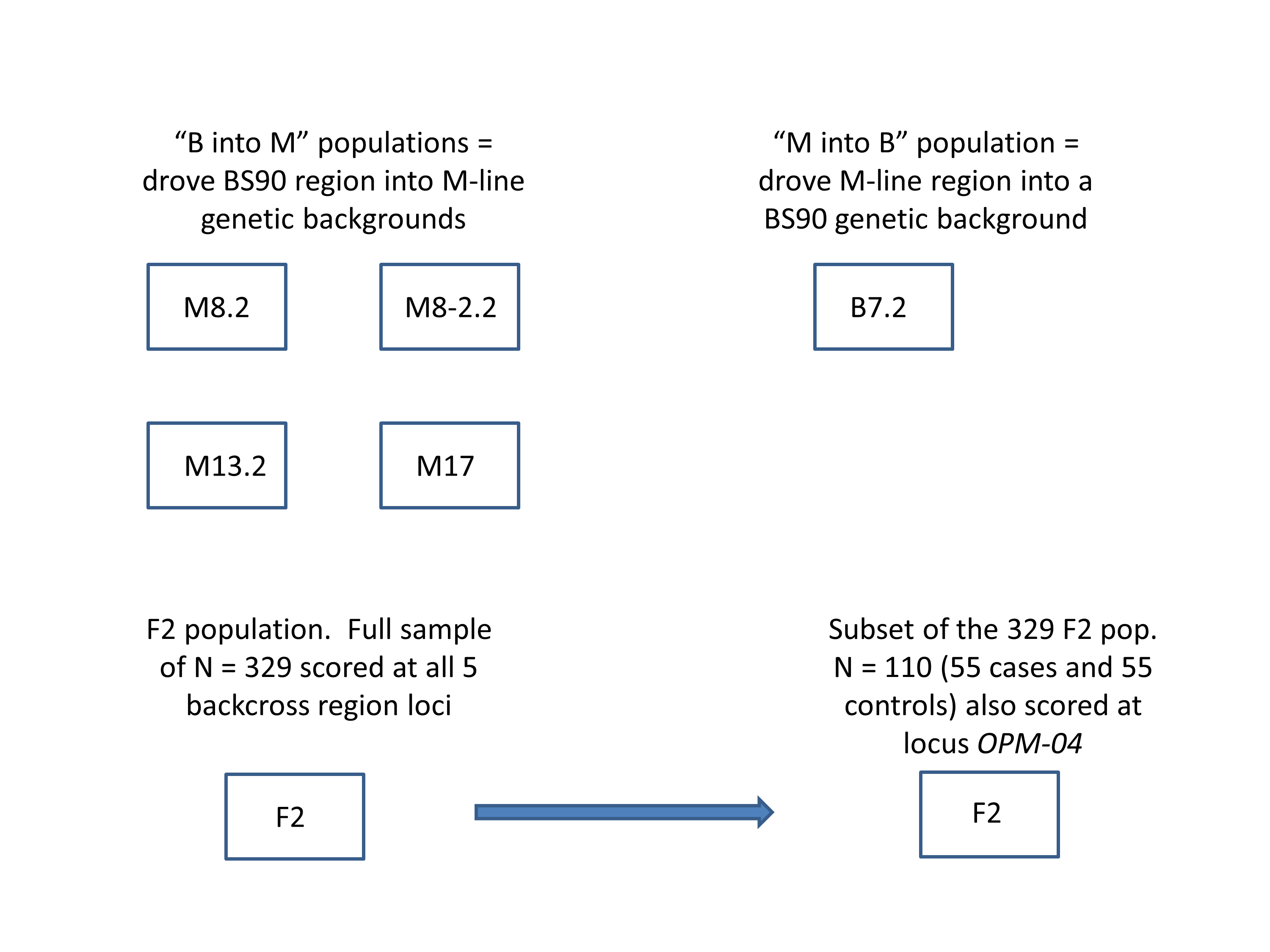

Supplement: Supplemental Information 5 [file peerj-10-13971-s005.png]

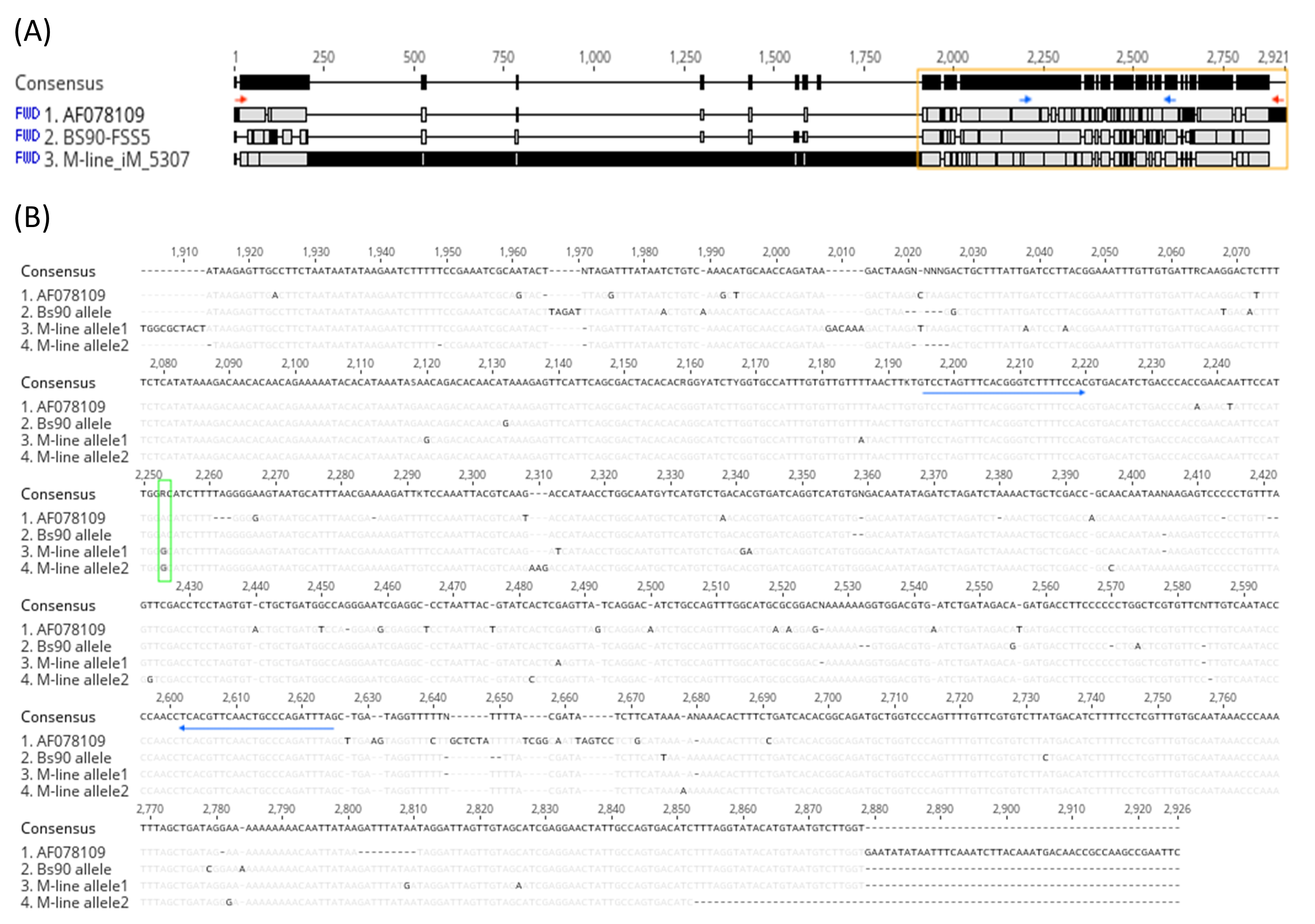

Supplement: Supplemental Information 6 — (A) Structure of the published sequence for OPM-04 (AF078109) and the homologous regions in our BS90 (BS90-FSS5) and M-line assemblies (M-line_iM_5307). Notice the ~1.7 kb insertion in the M-line assembly. Red arrows show the beginning and end of Knight et al. (1999) sequence. Blue arrows are primer positions for the region we sequenced to score variation in the F2 population. Orange box is the section of the alignment shown in (B). (B) Alignment of the part of the OPM-04 region downstream of the 1.7 kb insert. Blue arrows = the primers we used for sequencing. Green box = the A/G SNP we scored to distinguish the BS90 allele from the two M-line alleles in our F2 population. [file peerj-10-13971-s006.png]
